# Impact of Multidisciplinary Team Management on the Survival Rate of Head and Neck Cancer Patients: A Cohort Study Meta-analysis

**DOI:** 10.3389/fonc.2021.630906

**Published:** 2021-03-08

**Authors:** Changyi Shang, Linfei Feng, Ying Gu, Houlin Hong, Lilin Hong, Jun Hou

**Affiliations:** ^1^Department of Oral and Maxillofacial Surgery, The First Affiliated Hospital of Anhui Medical University, Hefei, China; ^2^Department of General Dentistry, School of Dental Medicine, Stony Brook University, Stony Brook, NY, United States; ^3^Program in Public Health, Stony Brook Medicine, Stony Brook University, Stony Brook, NY, United States; ^4^Department of General Dentistry, The Fourth Affiliated Hospital of Anhui Medical University, Hefei, China

**Keywords:** multidisciplinary team management, MDTM, head and neck cancer, HNC, survival rate, meta-analysis

## Abstract

**Background:** Head and neck cancer (HNC) is one of the more common malignant tumors that threaten human health worldwide. Multidisciplinary team management (MDTM) in HNC treatment has been introduced in the past several decades to improve patient survival rates. This study reviewed the impact of MDTM on survival rates in patients with HNC compared to conventional treatment methods.

**Methods:** Only cohort studies were identified for this meta-analysis that included an exposure group that utilized MDTM and a control group. Heterogeneity and sensitivity also were assessed. Survival rate data for HNC patients were analyzed using RevMan 5.2 software.

**Results:** Five cohort studies (*n* = 39,070) that examined survival rates among HNC patients were included. Hazard ratios (HR) were calculated using the random effect model. The results revealed that exposure groups treated using MDTM exhibited a higher survival rate [HR = 0.84, 95% CI (0.76–0.92), *P* = 0.0004] with moderate heterogeneity (*I*^2^ = 68%, *p* = 0.01). For two studies that examined the effect of MDTM on the survival rate for patients specifically with stage IV HNC, MDTM did not produce any statistically significant improvement in survival rates [HR = 0.81, 95% CI (0.59–1.10), *p* = 0.18].

**Conclusions:** The application of MDTM based on conventional surgery, radiotherapy, and chemotherapy improved the overall survival rate of patients with HNC. Future research should examine the efficacy of MDTM in patients with cancer at different stages.

## Introduction

Head and neck cancer (HNC) consists of a group of malignant neoplasias involving different anatomical regions, including the oral cavity, pharynx, larynx, paranasal sinuses, nasal cavity, and salivary glands (1). HNC is the sixth most common type of cancer among humans, and every year, over 650,000 HNCs are diagnosed worldwide, contributing to more than 330,000 deaths annually (2, 3). High rates have been reported on the Indian subcontinent and other parts of Asia, with male incidence rates exceeding 10 per 100,000 annually (4, 5). HNC presents with the characteristics of invasion and malignancy, and 90% of HNCs are squamous cell carcinoma (HNSCC) (6). Among the cases of HNSCC, oral squamous cell carcinoma (OSCC) comprises the majority of HNCs and accounts for approximately 90% of all oral malignancies (7). Although HNC usually is curable if diagnosed early, the lack of patient awareness of early warning signs makes early diagnosis challenging. About two-thirds of HNC patients already have advanced to stages III and IV at the time of diagnosis, leading to increased postoperative recurrence and metastasis (8, 9). The resulting poor prognosis leads to a 5-year survival rate of ~50% for HNC patients (10, 11).

To promote better cancer treatment outcomes, medical institutions have established multidisciplinary team management (MDTM). MDTM refers to the method of clinical diagnosis and treatment drawn from two or more related disciplines with the participation of representatives from each relevant medical specialty. The core activity of MDTM utilized to improve patient prognosis is to hold MDT meetings, at which all new cases of HNC are discussed, and each patient receives a personalized diagnosis and treatment plan (12). Also, patients undergoing surgery, radiotherapy, or chemotherapy for HNC are suggested to have weekly discussions for the duration of their treatment (13). MDTM integrates the professional knowledge associated with various disciplines and breaks down professional boundaries of these disciplines, resulting in improved diagnosis and treatment. The MDTM teams usually include a trained head and neck surgeon. In addition to medical oncology and radiation oncology, MDTM teams can include radiologists, speech therapy, nutritional experts, pathology, dental services, nurses, and social work (14). However, there is no international consensus concerning the necessary professional team members from participating disciplines to be included on MDTM teams established for HNC (15, 16).

The time consumption and financial burden of regular MDT meetings are high, and some researchers believe that the cost for MDTM exceeds its benefits (17, 18). For patients with HNC, one of the greatest benefits of MDTM is improved survival rates. Recently, researchers have explored the impact of the application of MDTM to conventional surgery, radiotherapy, and chemotherapy on patient survival rates, but the results are controversial (19–22). The therapeutic effect of MDTM in improving HNC outcomes has not been studied thoroughly. In this paper, it was hypothesized that MDTM improved the survival rate of patients with HNC.

## Methods

This meta-analysis study was prepared according to the Preferred Reporting Items for Systematic Reviews and Meta-Analyses (PRISMA) guidelines (23) and the Meta-analysis of Observational Studies in Epidemiology (MOOSE) guideline (24). It was conducted using the methodology recommended by the Cochrane Collaboration (25).

### Selection Criteria

Studies were included in data analyses if they met the following criteria. (1) The studies were cohort studies and published as original studies. (2) They assessed head and neck cancers with MDTM as an exposure and had conventional surgery, radiotherapy, or chemotherapy treatment measures as a control for comparison. (3) The studies analyzed survival rate as an outcome measure. (4) The studies used appropriate statistical analyses, such as hazard ratios and effect sizes or translatable data between the exposure and control groups.

### Search Strategy

PubMed, Cochrane, EMBASE, and Web of Science English databases were systematically searched for publications on MDTM of HNC patients. Searches were limited to articles published in English until January 2020. The main search terms included “head and neck squamous cell carcinoma,” “oral cancer,” “mouth tumor,” “nasopharyngeal tumor,” “sino-nasal tumor,” “pharyngeal tumor,” “laryngeal tumor,” “multidisciplinary team,” and “survival.” Titles, abstracts, and keywords were carefully examined to retrieve all relevant articles. In addition, the reference lists from the retrieved articles also were examined, and Medical Subject Headings (MeSH) were used to identify relevant studies.

### Data Extraction

Two reviewers (CS and LF) independently screened articles retrieved from databases using the inclusion criteria mentioned above. The full text of the articles was carefully reviewed, and data were extracted from each selected study. In cases of disagreement and inconsistencies, a third researcher (JH) was consulted for adjudication. For each study, the publication year, country, research type, sample size, exposure factors, and outcome measures were extracted.

### Quality Assessment

Currently, the Newcastle-Ottawa scale (NOS) is the most commonly used bias risk assessment tool for cohort studies (26). The NOS is divided into two parts, which are appropriate to evaluate cohort and case-control studies. Each part has three columns, including study population selection, comparability, and exposure or outcome evaluation, and eight items in total. The NOS bias risk was evaluated using a semi-quantitative star system, with a full score of nine stars. Two evaluators (CS and LF) evaluated the methodological quality for each cohort study included in this meta-analysis. Discrepancies were resolved when a consensus was reached with the third researcher (JH).

### Statistical Analysis

The effects of MDTM were presented using hazard ratios (HR) and 95% confidence intervals (CI). The study heterogeneity was evaluated using Chi-square tests or Q tests for *I*^2^ values. The *I*^2^ value of heterogeneity was categorized as no, small, moderate, and large heterogeneity with values of 0, 25, 50, and 75%, respectively. When the heterogeneity was small (P_Q_ ≥ 0.1 or *I*^2^ ≤ 50%), the combined HR and 95% CI were calculated using the Mantel-Haensel fixed-effect model. The Dersimonian-Laird random-effect model was used if the heterogeneity between studies was large (P_Q_ < 0.1 or *I*^2^ > 50%). The impact of individual studies on combined HR values was estimated using reassessment and missing mapping in each study. Subgroup analysis was performed to explore the source of heterogeneity. All analyses were performed using Review Manager (RevMan) version 5.2 (The Cochrane Collaboration, The Nordic Cochrane Centre, Copenhagen, Denmark) and Stata version 12.0 (StataCorp LP, College Station, TX, USA). *P*-values <0.05 were considered statistically significant.

## Results

### Search Results

**Figure 1:**
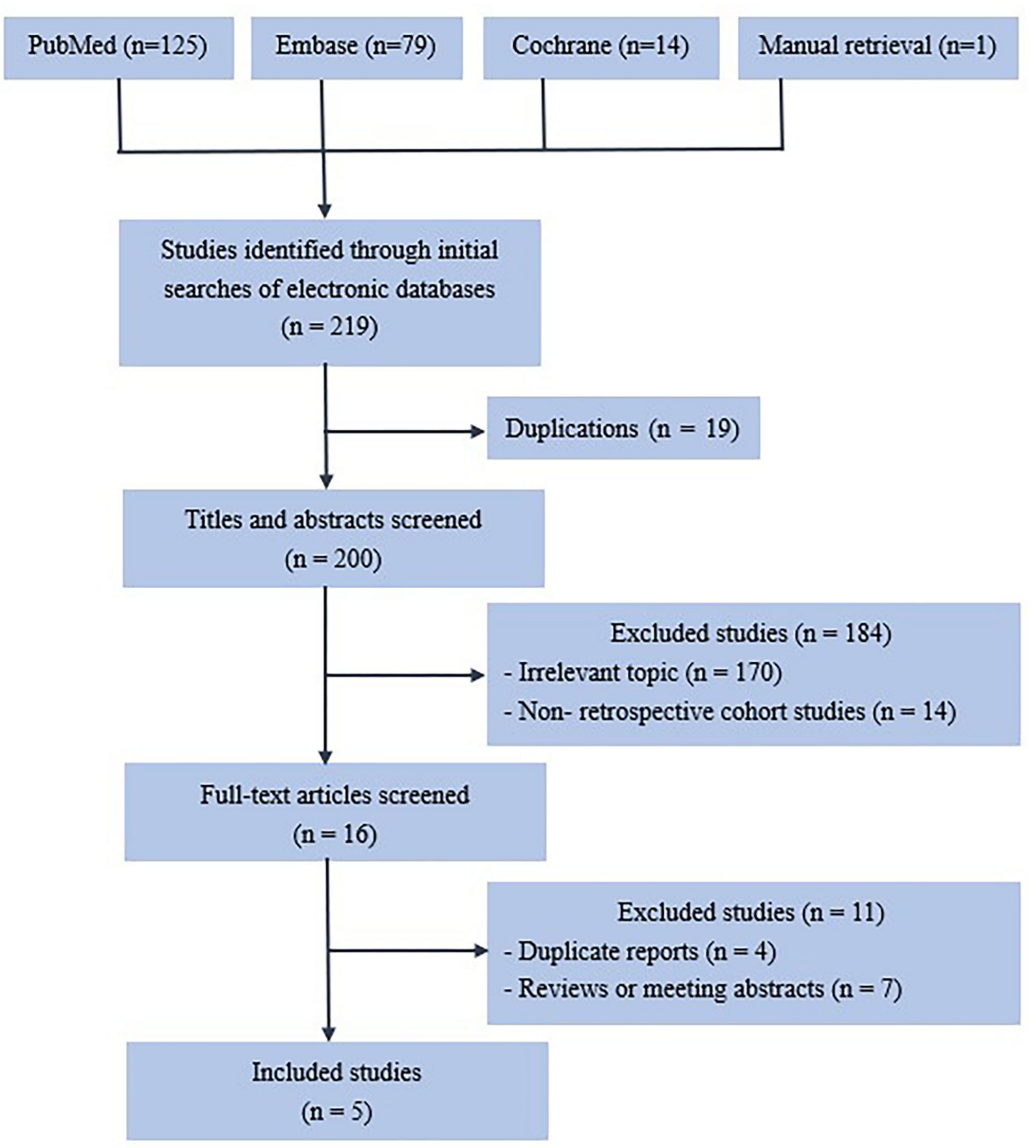
Flow chart showing the selection of papers.

Two hundred thirty-three articles were retrieved from the initial search (Figure 1), and 25 were removed due to duplication. A further 178 articles were excluded after the titles and abstracts were reviewed. Fourteen articles were excluded due to inappropriate research methods. Of the 16 remaining articles, four were excluded because of duplication, and seven failed to meet the inclusion criteria.

### Characteristics of the Eligible Studies

Five studies involving 39,070 patients were included in this meta-analysis (see Table 1). All five studies used HR as the outcome measure (13, 19, 20, 27, 28). The proportion of males ranged from 72 to 93.21%, and the average age ranged from 51 to 61.4 years. All five studies were adjusted for confounding effects (e.g., sex, age, race, disease stage, tumor location, hospital level, and others) to evaluate the association between MDTM and the survival of patients with HNC using survival models.

**Table 1:** Main characteristics of studies included in the meta-analysis.

**Study**	**Year**	**Country**	**Study type**	**Study time**	**Number**	**Study scope**	**Cancer stage**	**HR**	**95%CI**	***P***	**NOS score**
P. L. Friedland et al.	2011	Australia	Retrospective cohort study	1996–2008	726	H&N	I–IV	0.79	0.64–0.97	0.024	6
Y. H. Wang et al.	2012	Taiwan,China	Retrospective cohort study	2004–2008	19,513	Oral	-	0.84	0.78–0.90	0.001	7
W. C. Tsai et al.	2015	Taiwan,China	Nationwide cohort study	2004–2010	16,991	Oral	-	0.94	0.89–1.00	0.032	6
C. T. Liao et al.	2016	Taiwan,China	Retrospective cohort study	1996–2011	1,616	Oral	III–IV	0.75	0.63–0.89	0.001	5
J. C. Liu et al.	2019	America	Retrospective cohort study	2006–2015	224	H&N	I–IV	0.67	0.46–0.98	0.041	6

### Methodological Quality of the Included Studies

As shown in Table 2, the baseline consistency between the exposed group and the control group in each study was satisfactory and comparable. The median of the NOS quality evaluation for the five cohort studies was an average value of 6.00 ± 0.71 (range 5–7).

**Table 2:** NOS of studies included in the meta-analysis.

	**Study population selection**					**Result measurement**			
**Study**	**Exposure group representativeness**	**Control group selection method**	**Methods for determining exposure factors**	**Whether there are outcome indicators to be observed at the beginning of the study**	**Comparability between groups**	**Sufficiency of result evaluation**	**Length of follow-up time**	**Adequacy of follow-up**	**NOS score**
P. L. Friedland et al.	1	1	1	1	1	0	1	0	6
Y. H. Wang et al.	1	1	1	1	1	0	1	1	7
W. C. Tsai et al.	1	1	1	1	1	0	1	0	6
C. T. Liao et al.	1	1	0	1	1	0	1	0	5
J. C. Liu et al.	1	1	1	1	1	0	1	0	6

### Primary Outcome

#### The Effect of MDTM on the Survival Rate of Patients With HNC

**Figure 2:**
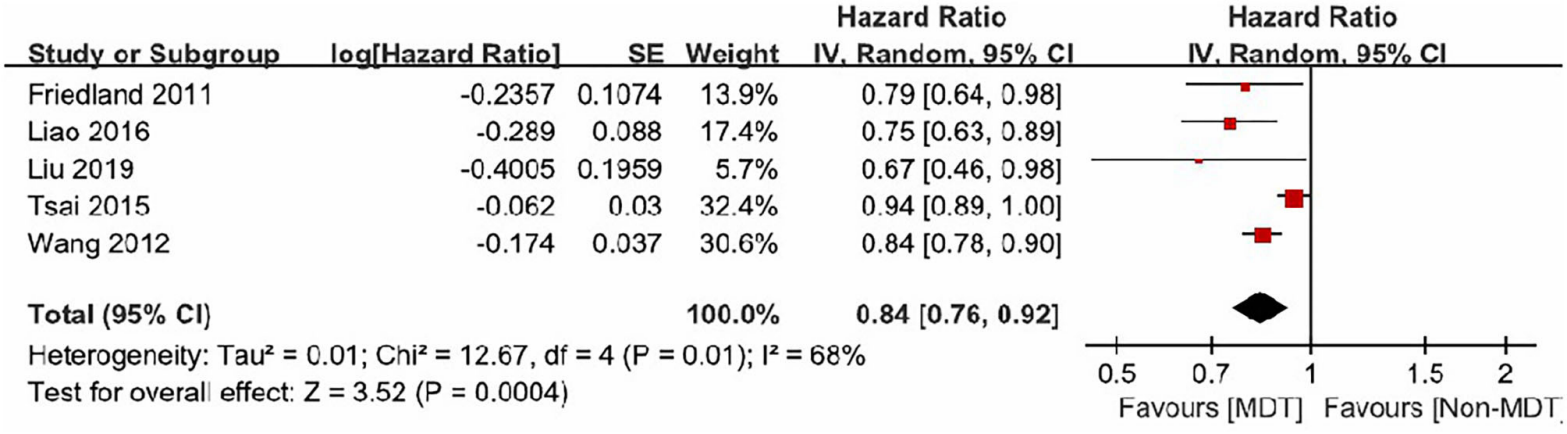
Forest plot of MDTM patients' survival rate.

The five studies included in this analysis demonstrated moderate heterogeneity (I^2^ = 68%, *p* = 0.01). Therefore, a random-effect model was used to estimate the MDTM treatment effect. The model suggested that MDTM resulted in a significantly higher survival rate in HNC patients compared to conventional methods [Overall HR: 0.84, 95% CI (0.76–0.92), *Z* = 3.52, *P* = 0.0004]. Thus, MDTM produced a 16% improvement in survival rate (Figure 2).

#### Meta-Analysis of the Effect of MDTM on the Survival Rate of Patients With Stage IV HNC

**Figure 3:**
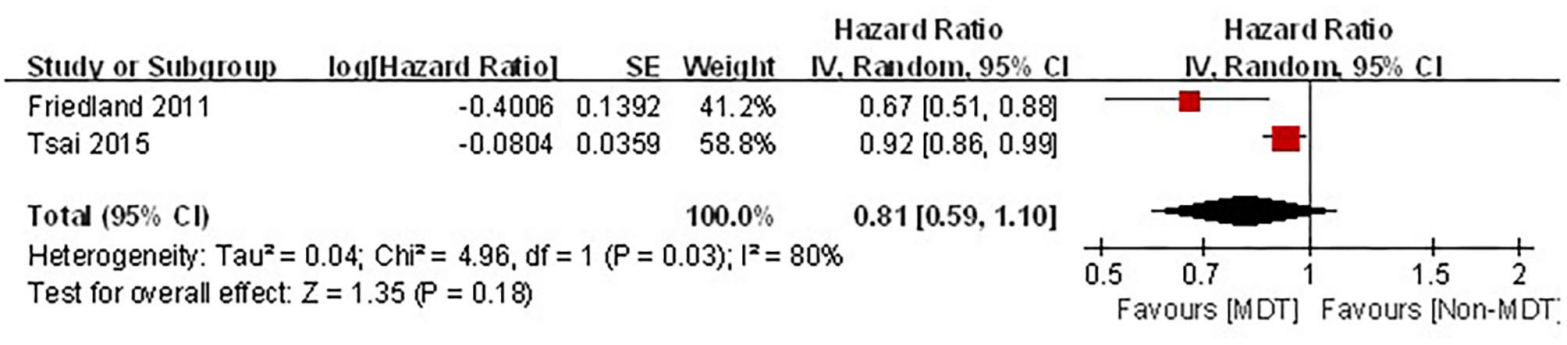
Forest plot of MDTM survival rate of stage IV cancer patients.

Of the five articles included in the analysis, two studies described the effects of MDTM management on the survival rate of patients with stage IV HNC (19, 20). The cancer stages were determined by the Union for International Cancer Control (UICC) cancer staging system in Friedland's study (19); while the American Joint Committee on Cancer (AJCC) cancer staging system was used in Tsai's study (20). Greater heterogeneity was observed between these two studies (*I*^2^ = 80%. *P* = 0.03). Although MDTM showed a trend toward an improved survival rate among patients with stage IV HNC, it did not reach statistical significance [combined HR = 0.81, 95% CI (0.59–1.10), *Z* = 1.35, *P* = 0.18, Figure 3].

### Sensitivity Analysis

**Figure 4:**
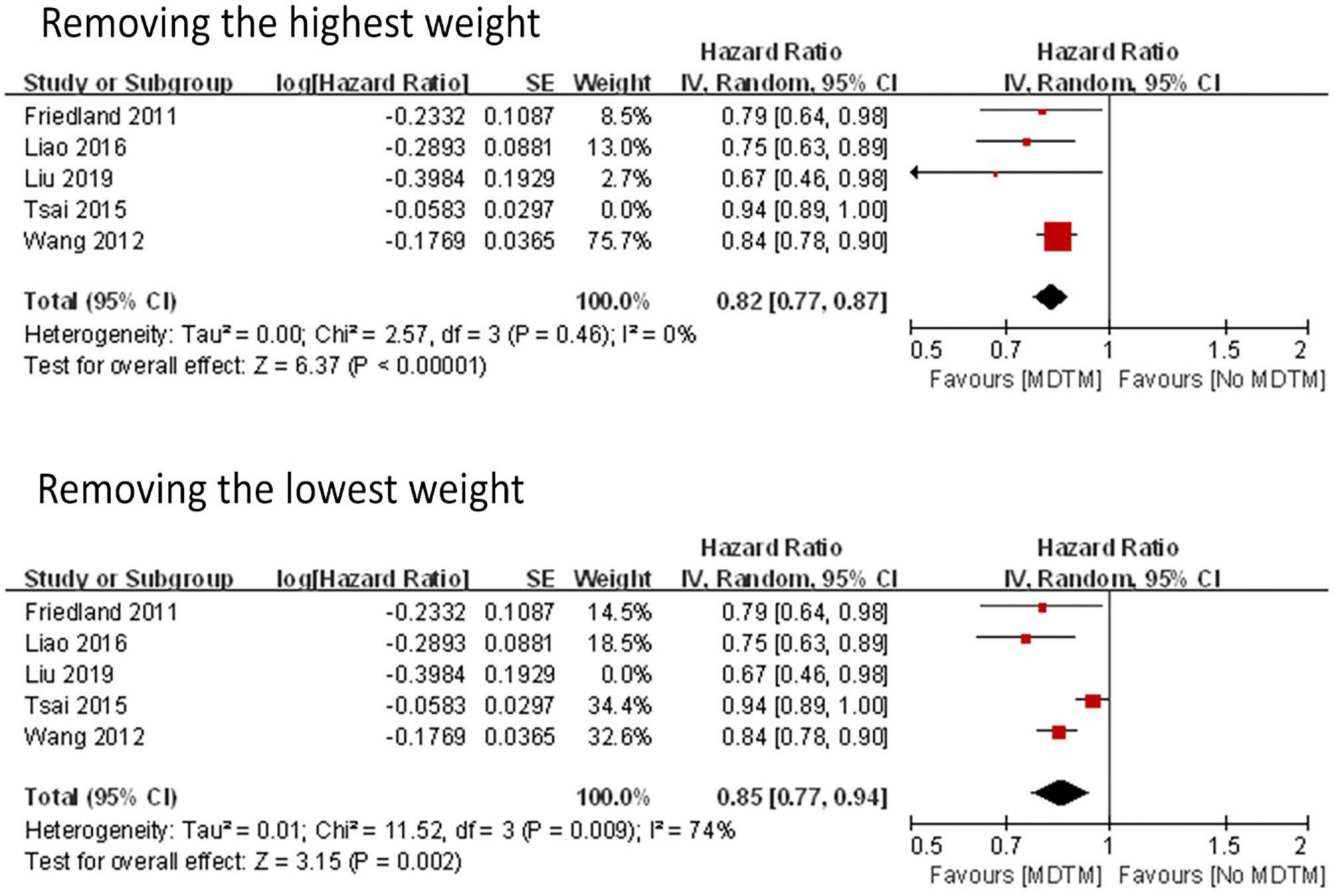
Forest plot of MDTM survival rate removing the highest and lowest weight.

Two models were used to assess sensitivity, including removing the highest gravity study (20) and removing the lowest gravity study (13) (Figure 4). The results of the two models were similar (HR with the removal of the highest gravity = 0.82; HR with the removal of the lowest gravity = 0.85). However, the heterogeneity reached zero when the highest gravity study was removed (*I*^2^ = 0%), indicating that the removed study was a major source of the heterogeneity (Table 3).

**Table 3:** Sensitivity Analysis.

**Analysis item**	***P***	***I*^**2**^**	**Effect model**	**HR**	**95%CI**
Remove the highest proportion of literature	0.44	0%	Fixed-effect model	0.82	0.77–0.87
Remove the lowest proportion of literature	0.01	72%	Random-effect model	0.85	0.77–0.94

### Subgroup Analyses

Subgroup analyses were performed according to the nationality of the study subjects to determine possible sources of heterogeneity. Three of the five studies (20, 27, 28) were from Asia, while the other two (13, 19) were conducted in Australia and the United States. The heterogeneity for studies from Asia was high (*I*^2^ = 81%, *p* = 0.005), while the heterogeneity for the other two countries was low (*I*^2^ = 0%, *p* = 0.47). HRs for studies from Asia and non-Asian countries were 0.86 and 0.76, respectively, (both *p*-values <0.01), and were not significantly different between Asia and non-Asian countries (*p* = 0.27).

## Discussion

Since HNC consists of a collection of complex and heterogeneous malignant tumors, it requires a range of treatment strategies. MDTM combines evidence-based treatment models, local experience, and well-developed management skills. To promote efficient and effective evidence-based management of HNC cases, most medical centers have established a process for MDTM that includes the participation of representatives from each relevant medical specialty. Treatment plans are made based on accurate tumor staging and other factors, including physical rehabilitation, mental health, and economic conditions that are tailored for different individuals in the MDTM meetings. A recent study evaluated multidisciplinary team meetings in a national tertiary referral center in Morocco and found that out of 105 patients (50.72%) who were scheduled for a MDTM meeting, 79 (38%) received and completed the MDTM meeting before treatment (29). According to the classification statistics for the different treatment methods for patients who were scheduled for a MDTM meeting, the proportion of patients who completed the MDTM meeting was 68% for surgery, 35% for medical treatment, and 19% for radiotherapy. Of the patients discussed at the MDTM meetings, 4–45% received changes in the post-meeting diagnostic reports, and they were more likely to receive more accurate and complete preoperative staging and new neo-adjuvant or adjuvant treatment (18).

We reviewed two retrospective studies that evaluated the role of MDTM in HNC. Nguyen et al. reviewed 225 patients with locally advanced HNC to identify how treatment outcomes were affected by MDTM recommendations. The authors concluded that MDTM approaches provided optimal treatment outcomes for locally advanced HNC (30). Birchall et al. found that patients assessed using MDTM experienced improved 2-year survival outcomes compared with patients who were not assessed using MDTM (*p* = 0.03) (31). The MDTM approach for treating patients with HNC has improved the organization of standard clinical guidelines, but this development has yet to translate into a demonstrable impact on survival (21). Croke et al. reported that articles showing that MDTM improved the prognosis of tumor patients have great heterogeneity after statistical analysis, so the relationship between MDTM and the prognosis of tumor patients is not clear (22). We found evidence that supported the concept that MDTM significantly influenced clinical decision-making and treatment recommendations. However, scant evidence suggested that MDTM improved patient outcomes. Because the relationship between MDTM and the survival rate of patients with HNC is still uncertain, we conducted this meta-analysis.

Based on the literature search, we did not find meta-analysis research on this specific topic. Therefore, this was the first meta-analysis to evaluate the influence of MDTM on the survival rate of HNC patients. We found that the survival rate of patients with HNC was positively correlated with the use of MDTM. Compared to conventional treatments, MDTM improved the survival rate of patients with HNC, with a combined-effect HR of 0.84. Through sensitivity analysis, we observed that the change in the estimated value of the combined effect quantity was not apparent when the highest gravity was removed and subsequently the lowest gravity. These observations indicated that the results of this meta-analysis were stable. However, after removing the highest proportion item, the heterogeneity disappeared, indicating that the eliminated study was a dominant source of heterogeneity. This study included five cohort studies. Therefore, this study might have been affected by a range of biases. Specifically, the overall management quality of the MDTM in the exposure group and the baseline consistency in the control group were affected, resulting in bias. Using subgroup analysis, we determined that the differences observed in the study scope where the research was conducted might have been the source of heterogeneity. There were no subgroup analyses of the HNCs for different stages in this study because most reports did not provide relevant data or lacked complete data to conduct such analyses. Other influencing factors, including gender, occupation, and use of tobacco and alcohol, also contributed to bias. Therefore, additional high-quality cohort studies are needed for large-scale meta-analysis to reduce bias and confirm the reliability of the above conclusions.

The advantages of MDTM are as follows. (1) MDTM is targeted to develop the best treatment, minimize misdiagnoses, and reduce the ineffective treatment of patients. (2) A reasonable treatment plan can be formulated by many experts using MDTM, which avoids inefficient and less effective treatment plans resulting from multiple referrals, repeated examinations, and treatment plan changes that often occur with the traditional treatment protocols. Clear, straightforward, and effective treatment plans can help produce emotional stability in patients that might improve their compliance with the treatment, which is conducive to a more positive outcome of the disease. (3) MDTM can avoid the need for patients to change departments numerous times. This continuity improves treatment and can shorten the time patients must wait for treatment, which also can help improve the prognosis. (4) MDTM enables multiple professional medical experts to consult on and discuss specific cases, which promotes communication and understanding between different departments. Such cooperation ensures the formulation and implementation of optimal treatment plans and facilitates the development of clinical and basic scientific research. This cooperation is critical to allow younger medical students to learn from each other and gain valuable information by participating in the MDTM meetings. (5) Finally, MDTM promotes the improvement of the hospital's overall treatment levels and the survival rates of tumor patients (32).

Among the five studies included in this meta-analysis, Tsai et al. reported that MDTM had a strong beneficial effect on the survival rate of stage IV patients but limited effects on stage I-III patients (20). Friedland et al. did not observe any significant differences in the 5-year survival rates between the MDTM group and the non-MDTM group for stage I–III patients, but the 5-year survival rate for stage IV patients in the MDTM group was significantly higher than the non-MDTM group (19). Although these two studies suggested that MDTM could improve the survival rate of patients with stage IV HNC, the results of this meta-analysis indicated that the impact of MDTM on the survival rate of patients with stage IV HNC was not clear.

There are only two published reports on stage IV HNC at present, which are not enough to prove the effectiveness of MDTM. The limited influence of MDTM on the survival rate of patients with stage IV HNC could be due to several reasons. (1) Patients with stage IV HNC are in the late stages of cancer, and their condition is more severe. The treatments in late-stage cancer are primarily palliative treatments, and the effects of treatment measures on patient survival rates are limited. (2) The survival rate of patients with stage IV HNC is affected by the physical resilience of patients and the degree of cancer metastasis. (3) The distribution of HNC stages is unique, with a distribution skewed toward stage IVA/B in regionally advanced stages (Table 4). The UICC stages IVA and IVB can be treated with the possibility of cure, whereas stage IVC is a metastatic disease that has spread to distant regions of the body. For stage IVC patients, oncologists only treat the metastatic disease and do not treat the primary lesions. It should be noted that among the five studies included in the current analysis, cancer stages were determined by AJCC cancer staging system in three studies and UICC cancer staging system (Sixth Edition) was used in one study. In all five studies, the authors categorized cancer stages from I to IV, however no further subcategorization within stage IV cancers were given.

**Table 4:** HNC Stage (UICC Version 6th).

Stage I	Tumor size and invasion: 2 cm or less in diameter, no invasion in adjacent tissues;
	Lymph node involvement: no;
	Distant organ involvement: no.
Stage II	Tumor size and invasion: larger than 2 cm in diameter but <4 cm in diameter, or has invaded an adjacent tissues;
	Lymph node involvement: no;
	Distant organ involvement: no.
Stage III	Tumor size and invasion: larger than 4 cm in diameter, or
	Lymph node involvement: no;
	Distant organ involvement: no.
Stage IVA	Tumor size and invasion: any size and invasion;
	Lymph node involvement: yes, 3–6 cm;
	Distant organ involvement: no.
Stage IVB	Tumor size and invasion: the space in front of the cervical spine tumor invasion,called the mediastinum between carotid artery or both lungs structures, such as the trachea and esophagus;or
	Lymph node involvement: yes, larger than 6 cm;
	Distant organ involvement: no.
Stage IVC	No matter the size of the primary tumor and lymph node involvement, distant organ involvement (distant metastasis)

Several limitations of this study should be acknowledged. First, though the overall sample size is large, the number of studies examined is small. Second, the confounding factors controlled in each study were different, which might result in estimation bias. Third, there might be differences in MDTM levels, which could be the source of heterogeneity observed in the research results. Because only five studies were included in this meta-analysis, a funnel plot could not be used to analyze the publication bias. Fifth, MDTs are a relatively recent (past decade) introduction to the management of HNC patients. Therefore, the improvement in survival might reflect the increase in HPV oropharyngeal cancer and the improved treatment of those patients rather than the MDTM itself. Thus, conclusions should be drawn with caution. The impact of MDTM on the survival rate of patients with stage IV HNC is not clear, and more research is needed.

## Conclusion

MDTM plays a prominent role in cancer treatment. We systematically evaluated the impact of MDTM on the survival rate of HNC patients. MDTM demonstrated a higher survival rate for HNC patients overall. This paper provided evidence for the successful application of MDTM in the treatment of HNC patients. Thus, MDTM is recommended in the treatment of HNC.

At present, there are few reports on the differences in survival rates for patients with different stages of HNC when MDTM was used. Although two studies claimed that the positive impact of MDTM on the survival rate of patients with stage IV HNC was greater than that of patients with stage I-III, the results of this meta-analysis did not demonstrate a statistical difference. Therefore, future research should focus on the difference of the effects of MDTM on the survival rate of HNC patients in different stages of the disease. This information would allow doctors and patients to judge the necessity of using MDTM, reduce unnecessary time and money invested by patients, and conserve valuable medical resources.

## Data Availability Statement

The original contributions presented in the study are included in the article/supplementary material, further inquiries can be directed to the corresponding author/s.

## Author Contributions

CS, LF, and JH conceived and designed the research. CS ran the model. LH contributed to materials. CS and LF searched and retrieved articles from database both also wrote the manuscript. LH, HH, and YG edited the paper. All authors contributed to the article and approved the submitted version.

## Conflict of Interest

The authors declare that the research was conducted in the absence of any commercial or financial relationships that could be construed as a potential conflict of interest.

## Abbreviations

MDTM: Multidisciplinary team management
HNC: Head and neck cancer
OSCC: oral squamous cell carcinoma.
